# Intravenous delivery of adipose tissue-derived mesenchymal stem cells improves brain repair in hyperglycemic stroke rats

**DOI:** 10.1186/s13287-019-1322-x

**Published:** 2019-07-17

**Authors:** Mari Carmen Gómez-de Frutos, Fernando Laso-García, Luke Diekhorst, Laura Otero-Ortega, Blanca Fuentes, Jukka Jolkkonen, Olivier Detante, Anaick Moisan, Arturo Martínez-Arroyo, Exuperio Díez-Tejedor, María Gutiérrez-Fernández, on behalf of RESSTORE consortium

**Affiliations:** 1 Department of Neurology and Stroke Center, Neuroscience and Cerebrovascular Research Laboratory, La Paz University Hospital, Neuroscience Area of IdiPAZ Health Research Institute, Autonoma University of Madrid, Paseo de la Castellana 261, 28046 Madrid, Spain; 2 Department of Neurology, University of Eastern Finland, Kuopio, Finland; 3 NeuroCenter, Kuopio University Hospital, Kuopio, Finland; 4 Neurology Department, Stroke Unit, Grenoble Hospital, Grenoble, France; 5 Grenoble Institute of Neurosciences, Inserm U1216, Grenoble Alpes University, Grenoble, France; 6 Cell Therapy and Engineering Unit, EFS Auvergne Rhône Alpes, Saint-Ismier, France

**Keywords:** Adipose tissue, Behavioral outcome, Brain repair, Experimental model, Hyperglycemia, Mesenchymal stem cells

## Abstract

**Background:**

Over 50% of acute stroke patients have hyperglycemia, which is associated with a poorer prognosis and outcome. Our aim was to investigate the impact of hyperglycemia on behavioral recovery and brain repair of delivered human adipose tissue-derived mesenchymal stem cells (hAD-MSCs) in a rat model of permanent middle cerebral artery occlusion (pMCAO).

**Methods:**

Hyperglycemia was induced in rats by the administration of nicotinamide and streptozotocin. The rats were then subjected to stroke by a pMCAO model. At 48 h post-stroke, 1 × 10^6^ hAD-MSCs or saline were intravenously administered. We evaluated behavioral outcome, infarct size by MRI, and brain plasticity markers by immunohistochemistry (glial fibrillary acidic protein [GFAP], Iba-1, synaptophysin, doublecortin, CD-31, collagen-IV, and α-smooth muscle actin [α-SMA]).

**Results:**

The hyperglycemic group exhibited more severe neurological deficits; lesion size and diffusion coefficient were larger compared with the non-hyperglycemic rats. GFAP, Iba-1, and α-SMA were increased in the hyperglycemic group. The hyperglycemic rats administered hAD-MSCs at 48 h after pMCAO had improved neurological impairment. Although T2-MRI did not show differences in lesion size between groups, the rADC values were lower in the treated group. Finally, the levels of GFAP, Iba-1, and arterial wall thickness were lower in the treated hyperglycemic group than in the nontreated hyperglycemic group at 6 weeks post-stroke.

**Conclusions:**

Our data suggest that rats with hyperglycemic ischemic stroke exhibit increased lesion size and impaired brain repair processes, which lead to impairments in behavioral recovery after pMCAO. More importantly, hAD-MSC administration induced better anatomical tissue preservation, associated with a good behavioral outcome.

**Electronic supplementary material:**

The online version of this article (10.1186/s13287-019-1322-x) contains supplementary material, which is available to authorized users.

## Background

Stroke is a significant public health issue and is the most common cause of death and disability worldwide [1]. To date, only intravenous thrombolysis (tPA) and mechanical thrombectomy have been shown to be effective in the acute phase of ischemic stroke. However, the narrow therapeutic window (< 4.5 h for tPA and < 6–24 h for endovascular treatment [2, 3]) limits its application to a small percentage of patients. Cell therapy is an interesting and promising approach in stroke research. Adipose tissue-derived mesenchymal stem cells (AD-MSCs), among others, have been shown to improve functional recovery, with an increase in markers related to brain repair in experimental animal models of stroke [4, 5].

Hyperglycemia is present in a significant proportion of patients without diabetes [6]. Various studies have demonstrated that more than 50% of acute stroke patients present hyperglycemia on admission which predicts higher mortality and morbidity [7–9]. In experimental animal models, hyperglycemia in acute stroke is associated with a poor outcome, exacerbating processes involved in ischemic brain injury [10, 11]. Despite the high percentage of patients with comorbidities, most of the preclinical models are performed on healthy and young animals. Moreover, the effects of comorbidities including hyperglycemia on therapeutic effects are poorly studied [12], although this aspect is emphasized in The Stem Cell Therapies as an Emerging Paradigm in Stroke (STEPS) recommendations [13, 14].

To our knowledge, this study is the first to investigate whether the MSC treatment could improve functional outcome post-stroke in rats, even in hyperglycemic conditions. Therefore, our aim was to assess the impact of hyperglycemia on infarct size and behavioral recovery in a rat model of permanent middle cerebral artery occlusion (pMCAO). We also sought to evaluate the effect of hyperglycemia on the therapeutic response to intravenous administration of human AD-MSCs (hAD-MSCs) in an experimental model of ischemic stroke.

## Materials and methods

### Ethics statement

The procedure was carried out at our Cerebrovascular and Neuroscience Research Laboratory, La Paz University Hospital, Madrid, Spain. All experiments were designed to minimize animal suffering of animals in compliance and approved by our medical school’s Ethical Committee for the Care and Use of Animals in Research (Ref. PROEX 249/15) according to the Spanish (RD 1201/2005 and RD53/2013) and European Union (EU) (86/609/CEE, 2003/65/CE, 2010/63/EU) rules. Experiments were conducted according to the ARRIVE guidelines for reporting animal research in terms of randomization, blinding, and statistical power (https://www.nc3rs.org.uk/arrive-guidelines).

### Cell culture protocol, characterization, and hAD-MSC isolation

**Fig. 1:**
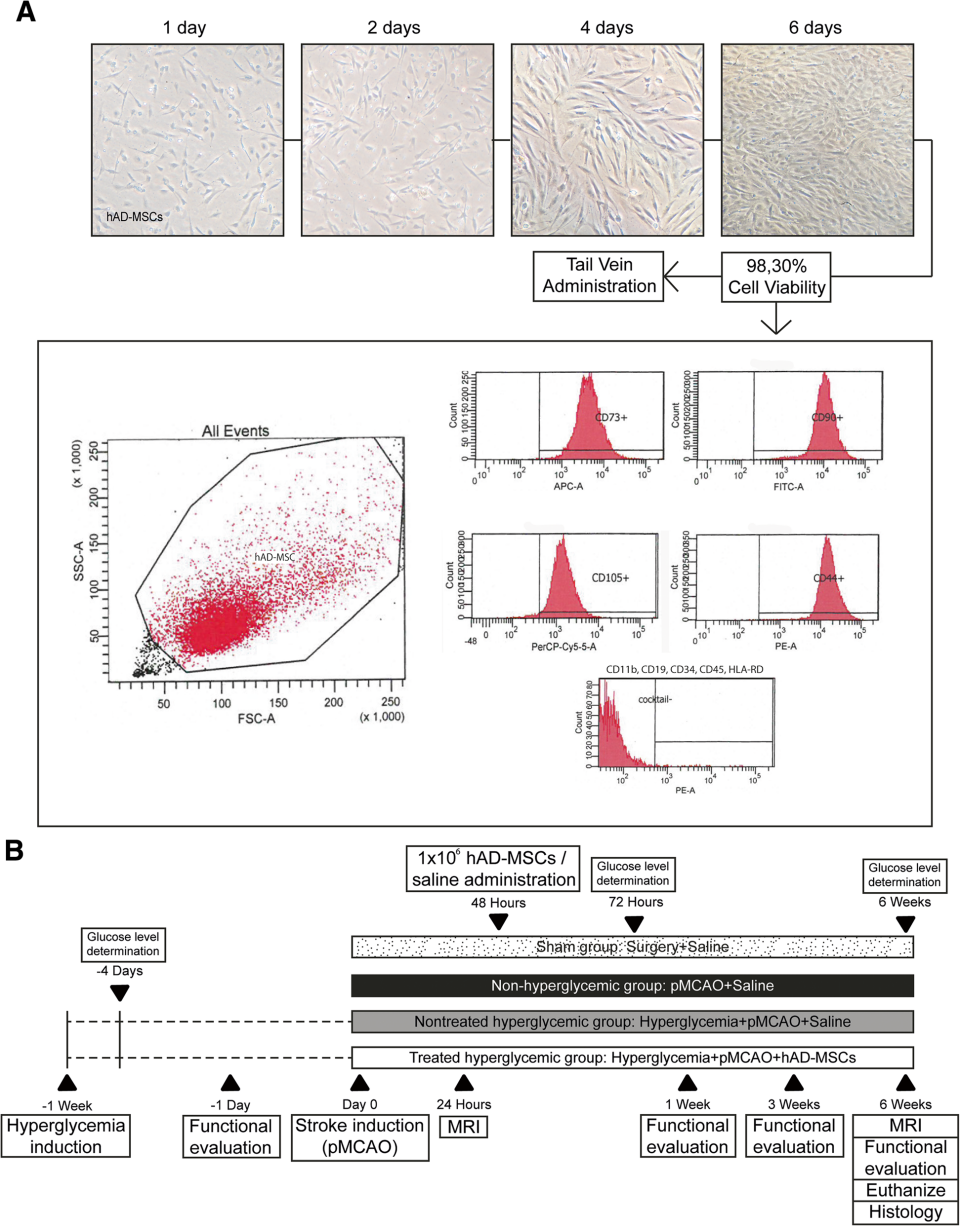
**a** Human AD-MSC cell culture protocol. Human AD-MSCs were thawed and cultured for their expansion on tissue culture flasks for 6 days. The phenotypic pattern of the cells was studied using flow cytometry, the positive expression of CD90, CD73, CD105 and CD44 (≥ 90%), and the lack of expression of CD11b, CD19, CD34, CD45, and HLA-DR were detected. Cell viability was studied and the cells were prepared for intravenous administration. **b** Experimental animal protocol. One week before stroke induction, hyperglycemia was induced in the rats with two intraperitoneal injections (nicotinamide and 15 min later streptozotocin). After 72 h and 6 weeks, blood glucose concentrations were determined. Rats were subjected to a cortical stroke by permanent middle cerebral artery occlusion and divided into groups. Forty-eight hours after surgery, 1 × 10^6^ hAD-MSCs or saline solution was administered. Functional evaluation was performed before surgery and at 1 week, 3 weeks, and 6 weeks post-stroke, and MRI was analyzed at 24 h and 6 weeks post-stroke. Six weeks post-stroke, the animals were euthanized and histological analyses were performed. Abbreviations: pMCAO: permanent middle cerebral artery occlusion; MRI: magnetic resonance imaging; hAD-MSCs: human adipose tissue-derived mesenchymal stem cells

The hAD-MSCs obtained from the French Blood Establishment (EFS, La Tronche, France) were cultured. The cells were thawed, expanded (using a seeding density of 2 × 10^3^ viable cells/cm^2^) on tissue culture flasks (Fisher Scientific), and maintained in Minimum Essential Media-alpha (1X) (MEM-Alpha, Gibco), supplemented with 5% PLTMax Human Platelet Lysate (Merck) and 1% penicillin/streptomycin with 5% CO_2_ at 37 °C. The phenotypic pattern of the cells was studied using flow cytometry, the positive expression of CD90, CD73, CD105, and CD44 (≥ 90%), and the lack of expression of CD11b, CD19, CD34, CD45, and HLA-DR were detected (Fig. 1a).

Cell viability was studied using 0.4% trypan blue (Trypan Blue solution, Sigma) and a Nikon Inverted Microscope Diaphot-TMD (Japan) with × 10 objective lens and a Nikon Phase Contrast-2 ELWD 0.3 Condenser (Japan). When the cells reached > 90% confluence, the hAD-MSCs were trypsinized (trypsin 0.25% ethylenediamine tetraacetic acid in Hanks’ Balanced Salt Solution [Biowest]) and centrifuged 10 min at 1250 rpm at room temperature. One million cells were resuspended in 1 ml saline for intravenous administration (Fig. 1a).

### Animals, hyperglycemia induction, and surgery

Hyperglycemia was induced using nicotinamide-streptozotocin. Streptozotocin has a nonspecific action in the body that causes organ deterioration due to its cytotoxic action [15, 16]. Nicotinamide was used to prevent organ damage [15]. In the present study, hyperglycemia was induced by an intraperitoneal injection with nicotinamide (210 mg/kg) (EMD Millipore, Germany) followed 15 min later with an intraperitoneal injection of streptozotocin (60 mg/kg) (EMD Millipore, Germany). After 72 h and again at 6 weeks, blood glucose concentration was determined using a glucose meter (ACCU-CHEK, Performa, Germany), and the animals with a blood glucose concentration above 250 mg/dl were considered hyperglycemic [17].

A total of 57 male Sprague-Dawley rats (8–9 weeks old, weighing 200–250 g) were used for the study. The rats were anesthetized via intraperitoneal injection of a solution of ketamine (25 mg/kg) and diazepam (2 mg/kg) at a dose of 2.5 ml/kg. Analgesia was induced by subcutaneous injection of meloxicam (2 mg/kg). To perform the pMCAO, a small craniotomy was performed; the right middle cerebral artery (MCA) was permanently ligated just before its bifurcation and both common carotid arteries were then occluded for 60 min as previously described [18].

The rats were randomly assigned to the following groups: the sham group was subjected to surgery without infarction (*n* = 10); the non-hyperglycemic group was subjected to a pMCAO and received intravenous saline (*n* = 10); the nontreated hyperglycemic group was subjected to a pMCAO and received intravenous saline (*n* = 10); and the treated hyperglycemic group was subjected to a pMCAO and received intravenous hAD-MSCs (*n* = 11). Saline solution or 1 × 10^6^ hAD-MSCs in 1 ml of saline solution was administered via the tail vein at 48 h after surgery. All the animals were euthanized at 6 weeks post-stroke (Fig. 1b).

### Functional evaluation scales

Functional evaluations were performed on all the rats by a blind observer before surgery and at 1 week, 3 weeks, and 6 weeks post-stroke induction. Motor and sensory performance was evaluated using the Rogers, beam walking, and adhesive removal tests. A variant of Rogers’ functional scale was used to assign scores as follows: 0, no functional deficit; 1, failure to extend forepaw fully; 2, decreased grip of forelimb while tail gently pulled; 3, spontaneous movement in all directions, contralateral circling only if pulled by the tail; 4, circling; 5, walking only when stimulated; 6, unresponsive to stimulation with a depressed level of consciousness; and 7, dead [19]. The beam walking test evaluated hindlimb functions [20] by the capacity of the rats to traverse a wooden beam. We calculated the left hind limb slip ratio as follows: (total slips + 0.5 × half slips)/total steps × 100% [21]. To assess the forelimb sensory asymmetry, the adhesive removal test was performed [20]. For this test, a sticker was placed on the palm of both forelimbs of the rat and contact and removal times were recorded [22].

### In vivo magnetic resonance imaging

Lesion size was analyzed at 24 h and 6 weeks post-stroke by magnetic resonance imaging (MRI) (Bruker Pharmascan, Ettlingen, Germany); 7-T horizontal bore magnets using T2-weighted (T2-W) spin-echo anatomical images acquired with a rapid acquisition with relaxation enhancement (RARE) sequence in axial orientations and the following parameters: two echo images (TE, 29.54 ms and 88.61 ms); TR = 3000 ms; RARE factor = 4; Av = 3; FOV = 3.5 cm; acquisition matrix = 256 × 256 corresponding to an in-plane resolution of 137 × 137 μm^2^; slice thickness = 1.00 mm without gap; and number of slices = 16.

We used diffusion-weighted imaging (DWI) including apparent diffusion coefficient (ADC) maps. Images were obtained with three different directions defined by the read, phase, and slice encoding gradients using a multi-shot spin-echo echo planar imaging (EPI) sequence. Acquisition conditions were diffusion gradient duration, 3 ms; diffusion gradient separation, 18 ms; TR, 3000 ms; TE, 50 ms; FOV, 3.8 cm; axial slices (1.5 mm thickness) and 3 *b* values 100, 400, and 1000s/mm^2^; acquisition matrix = 128 × 128. To normalize the ADC values, the ROI of the lesion and the same ROI in the contralateral side were divided by the value in the contralateral normal hemisphere and expressed as a relative ADC (rADC) of the region [23].

### Hematoxylin and eosin (H&E) staining

The histopathological changes in the cortex were studied by H&E staining. Slices were immersed 10 s in hematoxylin and 1 min in eosin. Finally, they were dehydrated and coverslipped with DePex. The multipolar motor neurons were observed using a × 20 objective lens and processed by image analysis software (Image-Pro Plus 4.1, Media Cybernetics) (3 rats for each group, 4 sections in each rat per group). Cell counts were expressed as individual values and as the mean number of viable neurons/mm^2^ [24].

### Immunohistochemistry and immunofluorescence

The perilesional area around the lesion core was defined on brain sections at 6 weeks post-stroke by microtubule-associated protein 2 (MAP-2) staining (1:1000, Millipore) and glial fibrillary acidic protein (GFAP) staining (1:500, Millipore) (Additional file 1: Figure S1). The samples were sectioned at 10 μm thickness using a Leica CM1950 cryostat (Leica). Immunohistochemistry images were obtained using a × 20 and × 40 objective lens, and processed by image analysis software (Image-Pro Plus 4.1, Media Cybernetics).

The perilesional area was studied in detail using immunofluorescence for astrocytes with GFAP (1:500, Millipore); microglia with Iba-1 (1:1000, Millipore); synaptic plasticity with synaptophysin (1:200, Sigma); neurons with doublecortin (1:250, Santa Cruz); endothelium with platelet endothelial cell adhesion molecule-1 (CD-31) (1:50, Abcam), collagen-IV (1:400, Abcam), and alpha-smooth muscle actin (α-SMA) (1:200, Abcam), followed by goat anti-mouse and anti-rabbit Alexa Fluor 488 (1:750, Invitrogen). Immunofluorescence images were acquired as a confocal maximum projection using a Leica TCS-SPE confocal microscope (Leica Microsystems, Heidelberg, Germany), using a × 40 objective lens, and analyzed using LAS AF software (Leica). Mean fluorescence intensity was measured by the NIS-Element AR (Nikon) 4.5 Program. The experiments, images, and quantification of the samples were performed by blinded observers using the same microscope configurations to eliminate bias due to background normalization (3 rats for each group, 4 sections in each rat per group).

### Statistical analysis

The results were expressed as mean ± standard deviation (SD), and the data were compared using Kruskal–Wallis test followed by the Mann–Whitney *U* test as the data followed a non-normal distribution. Values of *p* < 0.05 were considered significant at a 95% confidence interval; the data were calculated using the IBM SPSS statistical program 22 and GraphPad Prism 7 software. The rats removed from the study were immediately replaced by new subjects that were randomly allocated to the experimental groups until a total number of 10 rats per group was reached. The power analysis showed that with nonparametric testing for infarct size and behavioral tests, at least 10 rats needed to be randomized to each group for a significance level of 5% (alpha) and a power of 80% (1-beta).

## Results

### Mortality

Sixteen rats were excluded from the study: 13 died after surgical induction of permanent middle cerebral artery occlusion (pMCAO) (ten from the hyperglycemic group and three from the nonhyperglycemic group) and three were excluded because they did not show lesions on MRI analysis.

### Hyperglycemia-associated stroke-induced impairment of motor function

**Fig. 2:**
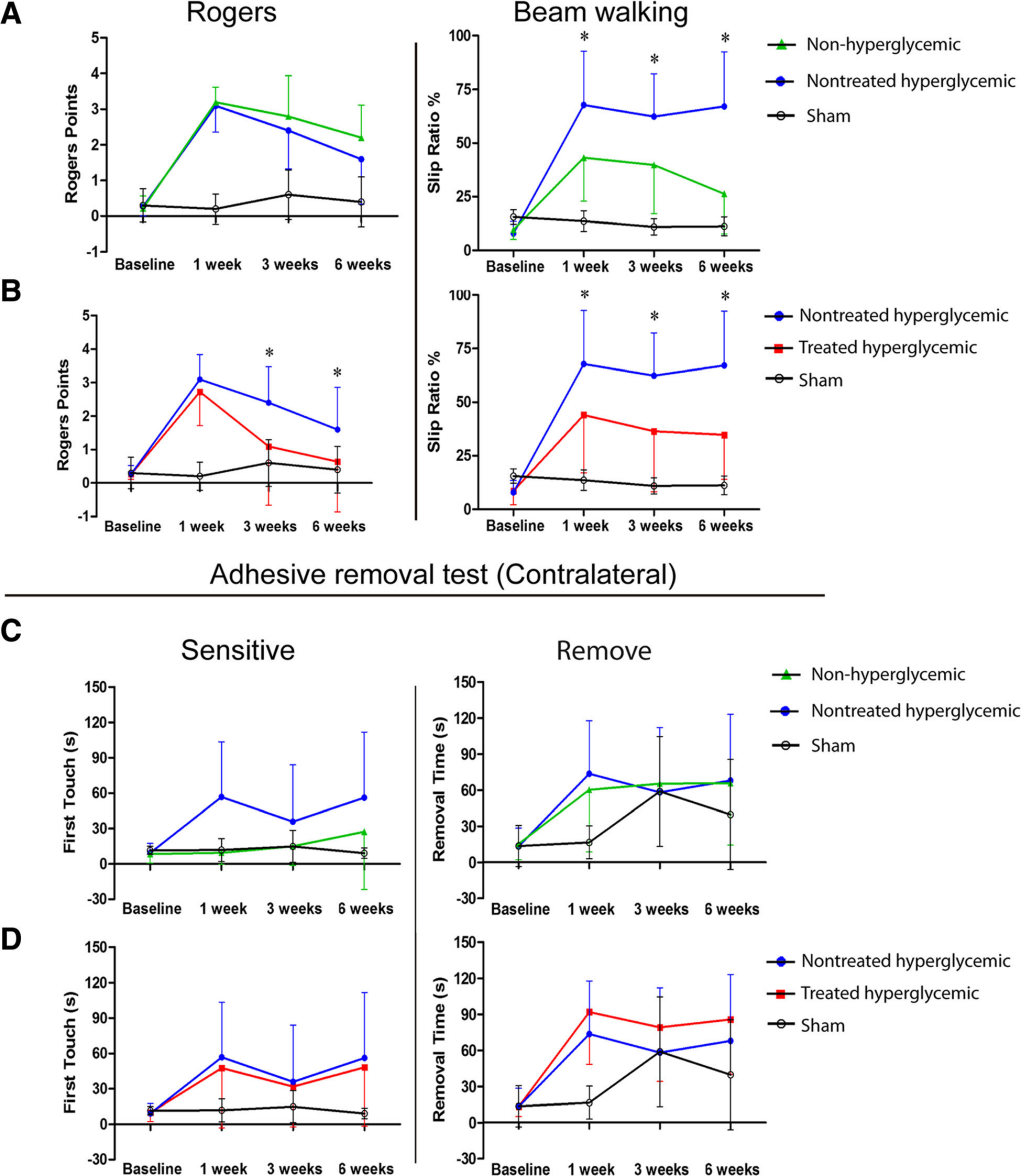
Functional evaluation of motor and sensory performance. Representative graphs of the functional evaluation tests: Rogers, beam walking (**a**, **b**) and adhesive removal (**c**, **d**) tests before stroke induction (baseline) and at 1 week, 3 weeks, and 6 weeks after permanent stroke. The sham-operated rats did not have any functional deficits. The hyperglycemic rats had poorer functional outcome than the non-hyperglycemic in the beam walking test at 1, 3, and 6 weeks post-stroke. The treated hyperglycemic rats showed better functional recovery compared with the hyperglycemic group without treatment in the Rogers test at 3 and 6 weeks and the beam walking test at 1, 3, and 6 weeks post-stroke (*n* = 10 animals per group). Data are shown as mean ± SD. *p < 0.05

The hyperglycemic group exhibited more severe neurological deficits, measured by the beam walking test at 1, 3, and 6 weeks post-stroke, compared with the non-hyperglycemic group (*p* < 0.05). The Rogers and adhesive removal tests showed no significant differences between the non-hyperglycemic and the hyperglycemic groups at all study times (*p* > 0.05) (Fig. 2a, c).

### hAD-MSC treatment significantly improved post-stroke neurological outcome in hyperglycemic rats

hAD-MSC treatment after pMCAO in hyperglycemic rats significantly improved neurological recovery compared with the nontreated hyperglycemic group, as indicated by the Rogers (at 3 and 6 weeks) and walking beam (at 1, 3, and 6 weeks) tests (*p* < 0.05).

The adhesive removal test showed no significant differences between the nontreated hyperglycemic and the treated hyperglycemic groups (*p* > 0.05) (Fig. 2b, d).

There were no significant differences in blood glucose level between non-treated hyperglycemic rats (before MCAO 351.75 ± 73.35 mg/dl; before euthanasia 445.0 ± 42.42 mg/dl) and treated hyperglycemic rats (before MCAO 368.5 ± 70.76 mg/dl; before euthanasia 408.0 ± 51.50 mg/dl) *P* > 0.05. It can be inferred that hAD-MSC treatment induces recovery which is not related to changes in blood glucose levels.

### Hyperglycemia significantly increased lesion size and diffusion coefficients after stroke in rats

**Fig. 3:**
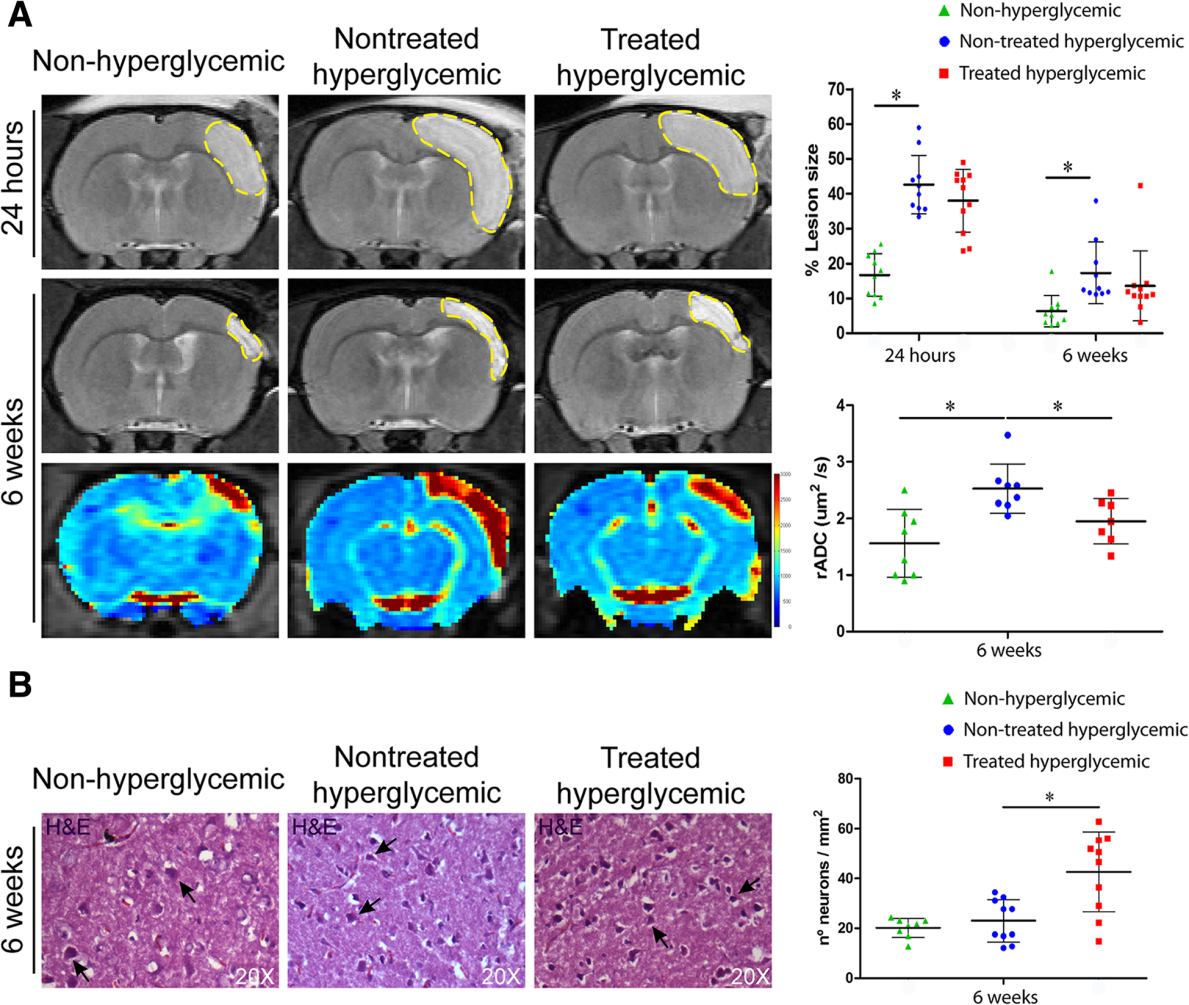
**a** MRI analysis. Representative T2-weighted MRI at 24 h and 6 weeks and rADC images at 6 weeks post-stroke. At 24 h and 6 weeks post-stroke, quantitative graphs of T2-weighted MRI showed a significantly higher lesion size in the hyperglycemic group compared with the non-hyperglycemic group. At 6 weeks post-stroke, the non-hyperglycemic and the treated hyperglycemic groups in the rADC images showed lower diffusion coefficients compared with the hyperglycemic rats (*n* = 10 animals per group). **b** Histopathological analysis. Representative H&E staining images in the cortex at 6 weeks. A significant increase in the number of surviving neurons was observed in the hyperglycemic treated group compared to the nontreated hyperglycemic group. (3 animals per group, 4 sections in each animal per group). Data are shown as mean ± SD. *p < 0.05. Abbreviations: MRI: magnetic resonance imaging; rADC: relative apparent diffusion coefficient; H&E: hematoxylin and eosin

The lesion size of the hyperglycemic group was significantly higher than that of the non-hyperglycemic group at 24 h (*p* < 0.001) and 6 weeks (*p* < 0.04) (Fig. 3a).

The rADC analysis showed significantly higher diffusion coefficients in the hyperglycemic rats compared with the non-hyperglycemic (*p* < 0.04) at 6 weeks post-stroke (Fig. 3a).

Hyperglycemia had no effect on the number of surviving neurons compared to non-hyperglycemic rats (*p* > 0.05) (Fig. 3b).

### hAD-MSC treatment did not reduce lesion size but significantly decreased diffusion and increased the number of surviving neurons post-stroke in hyperglycemic rats

At 24 h and 6 weeks, hAD-MSC treatment in the hyperglycemic rats did not decrease lesion size compared with the nontreated hyperglycemic group (*p* > 0.05) (Fig. 3a).

At 6 weeks post-stroke, the rADC values were higher in the nontreated hyperglycemic rats than in the treated hyperglycemic group (*p* < 0.03) (Fig. 3a).

The treated hyperglycemic group showed an increased in the number of surviving multipolar motor neurons compared with the nontreated hyperglycemic group (*p* < 0.003) (Fig. 3b).

### Hyperglycemia contributed to increased astrocyte and microglia markers

**Fig. 4:**
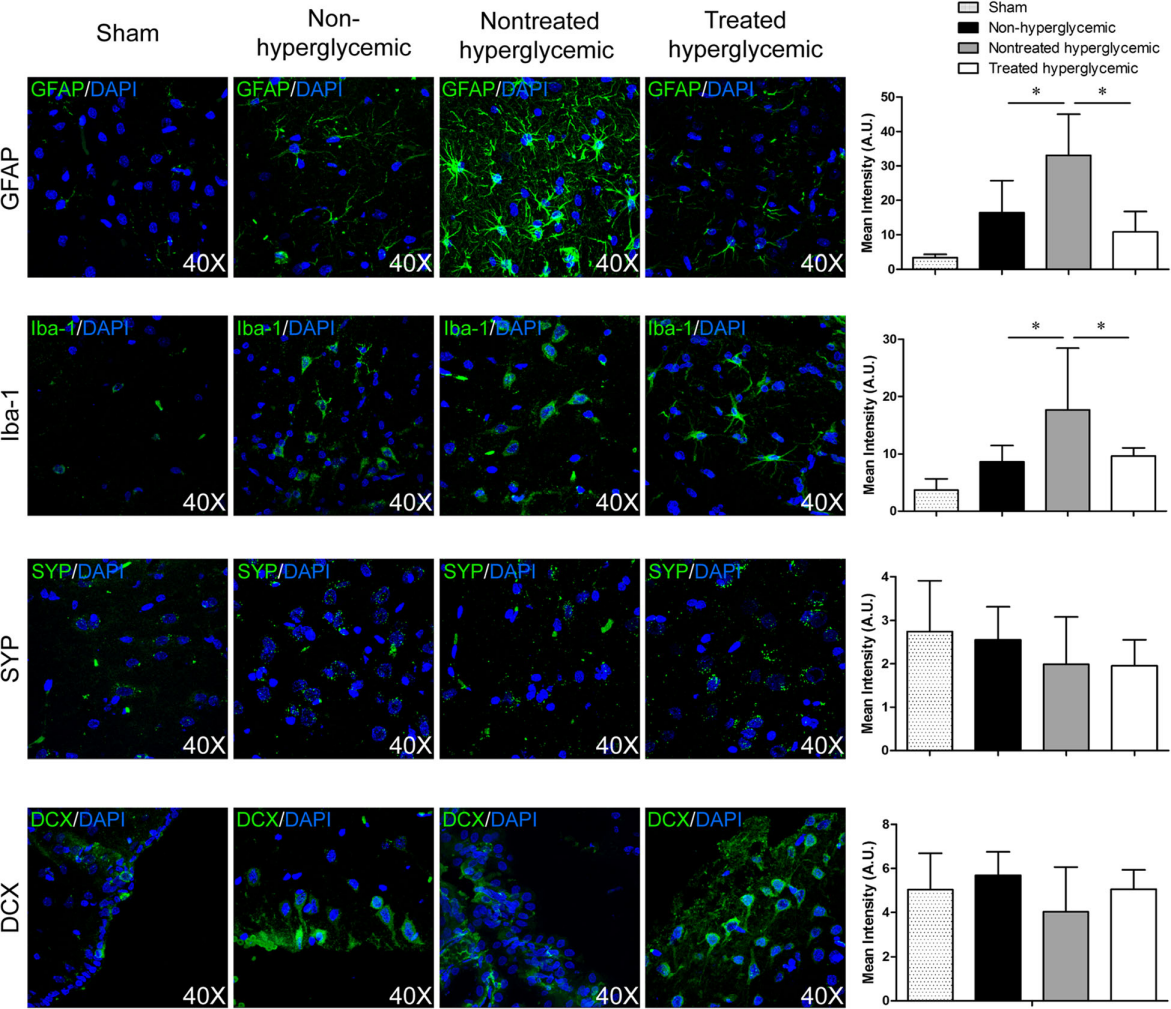
Brain repair markers related to glia and neurons at 6 weeks post-stroke by immunofluorescence. Representative images and quantification of immunofluorescence of GFAP, Iba-1, synaptophysin, and doublecortin. At 6 weeks post-stroke, the hyperglycemic rats showed significantly higher levels of GFAP and Iba-1 compared with the non-hyperglycemic and treated hyperglycemic groups. 4′,6-Diamidino-2-phenylindole (DAPI) was used for nuclear staining (3 animals per group, 4 sections in each animal per group). Data are shown as mean ± SD. *p < 0.05. Abbreviations: GFAP: glial fibrillary acidic protein; SYP: synaptophysin; DCX: doublecortin

There were increases in GFAP (*p* < 0.03) and Iba-1 (*p* < 0.012) levels in the hyperglycemic group compared with the non-hyperglycemic group (Fig. 4).

### hAD-MSC treatment decreased astrocytes and microglia post-stroke in hyperglycemic rats

To evaluate the effect of hAD-MSC treatment on glial cells, brain tissues were stained for the astrocyte marker GFAP and the microglia marker Iba-1. The expression of GFAP (*p* < 0.001) and Iba-1 (*p* < 0.03) was lower in the treated hyperglycemic group than in the nontreated hyperglycemic group at 6 weeks post-stroke (Fig. 4).

### Hyperglycemia induction has no effect on synaptogenesis and neurogenesis

The non-hyperglycemic rats did not show differences in the expression of synaptophysin and doublecortin compared with the hyperglycemic rats (*p* > 0.05) (Fig. 4).

### hAD-MSC treatment did not promote synaptogenesis and neurogenesis post-stroke in hyperglycemic rats

No differences were found between the hyperglycemic and treated rats in relation to synaptophysin and doublecortin staining at 6 weeks post-stroke (*p* > 0.05) (Fig. 4).

### Hyperglycemia had an effect on α-SMA, but not on vascular markers CD31 and collagen-IV

**Fig. 5:**
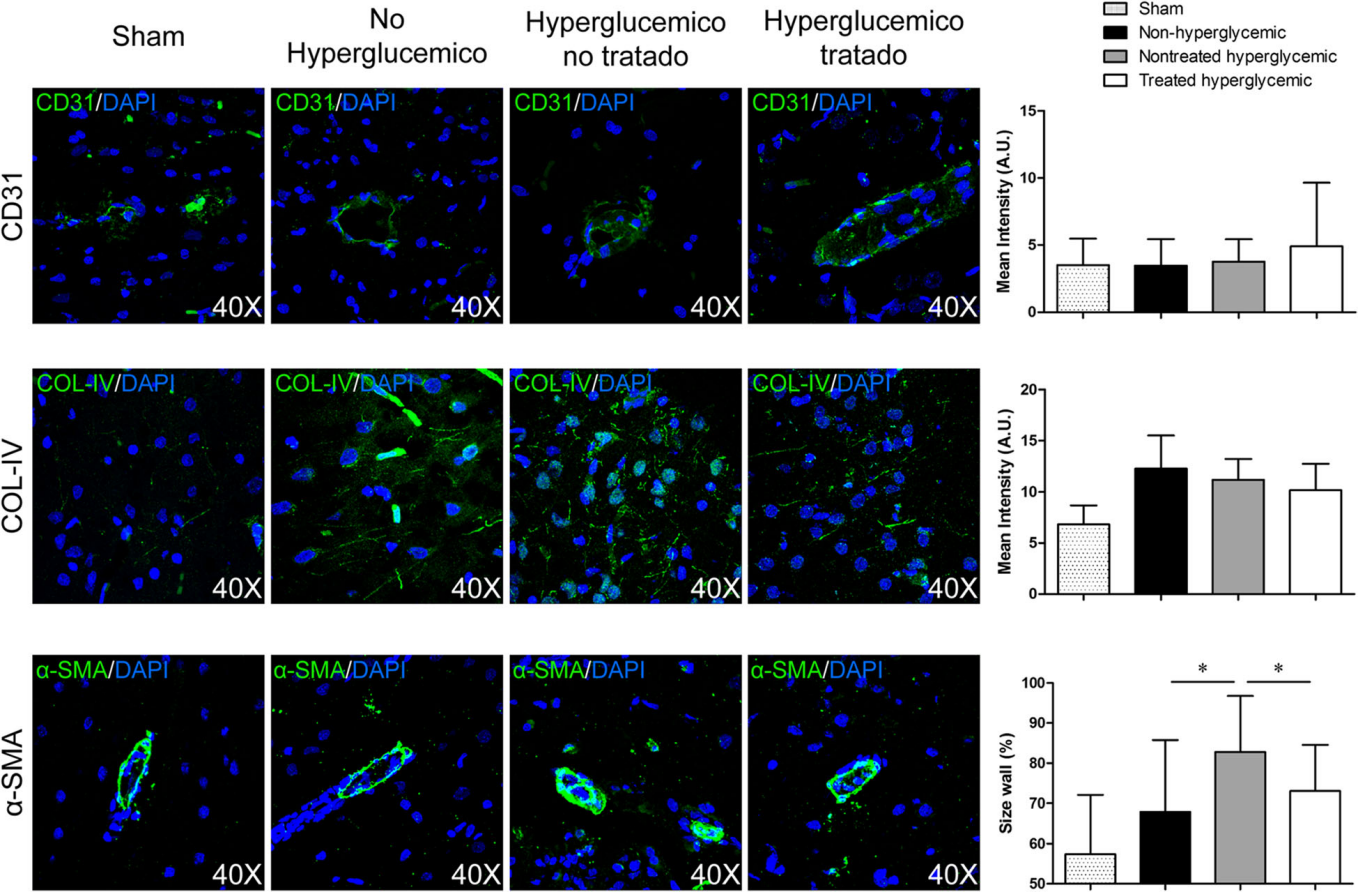
Vascular markers at 6 weeks post-stroke by immunofluorescence. Representative images and quantification of immunofluorescence of CD31, collagen-IV, and α-SMA. At 6 weeks post-stroke, the hyperglycemic rats showed significantly increased arterial wall thickness compared with the non-hyperglycemic and treated hyperglycemic groups. 4′,6-diamidino-2-phenylindole (DAPI) was used for nuclear staining (3 animals per group, 4 sections in each animal per group). Data are shown as mean ± SD. *p < 0.05. Abbreviations: α-SMA: α-smooth muscle actin

We found no differences in the expression of CD31 and collagen-IV markers between the non-hyperglycemic and hyperglycemic rats (*p* > 0.05). However, we found that hyperglycemia significantly increased α-SMA compared with the non-hyperglycemic group (*p* < 0.004) (Fig. 5).

### hAD-MSC treatment did not promote the formation of blood vessels but decreased arterial wall thickness

Our results suggest that the treatment did not promote blood vessel reconstruction in terms of CD-31 and collagen-IV markers compared with the hyperglycemic group (*p* > 0.05). However, we observed that α-SMA significantly decreased in the treated hyperglycemic rats compared with the hyperglycemic group (*p* < 0.015) (Fig. 5).

## Discussion

To date, the majority of the experimental animal studies of stroke associated with hyperglycemia have been performed using ischemia/reperfusion models. However, a high percentage of patients do not recanalize. Thus, it is important to introduce permanent ischemia models in order to understand the exact mechanisms by which hyperglycemia leads to poorer functional outcomes, as well as to continue investigating therapies that have demonstrated efficacy in stroke models without associated comorbidities.

Previous studies from our group have demonstrated that AD-MSC administration improves functional recovery, decreased cell death and increased brain plasticity markers after pMCAO [5, 25]. However, the present study is the first to investigate whether AD-MSC treatment could improve the functional outcome post-stroke in hyperglycemic rats, a comorbidity that remains relatively under explored.

It has been shown that hyperglycemia worsens damage probably by enhancing the production of lactic acid during ischemia. Ischemia blocks or retards the stage of pyruvate oxidation and leads to the reduction of pyruvate to lactate. The net result of the anaerobic metabolism of glucose is the production of lactate- and of H^+^. Thus, the enhancement of glucose supply by hyperglycemia may exaggerate the acidosis. The adverse effects of acidosis may promote edema formation by inducing Na^+^ and Cl^−^ accumulation via coupled Na^+^/H^+^ and C1^−^/HCO3^−^ exchange. Thus, the severity of the acidosis and exacerbated brain damage correlates to the preischemic hyperglycemia. This brain injury implicates damage to neurons, glial cells, and/ or vascular endothelium as well [26]. Our study confirmed that hyperglycemic ischemic rats exhibit an increased lesion size and impaired brain repair processes, and demonstrated an increase in the inflammatory response (Iba 1 and GFAP) and in the thickness of the arterial wall (α-SMA), which likely contribute to exacerbated impairments of behavioral recovery.

We also evaluated the repair effect of intravenous delivery of hAD-MSCs in a rat model of pMCAO associated with hyperglycemia. Our results demonstrated that hAD-MSC treatment induces lower diffusion coefficients with a decrease in the inflammatory response and in the thickness of the arterial wall, which is associated with a good behavioral outcome.

Stroke patients are a heterogeneous population that has associated comorbidities, such as hyperglycemia. Over 50% of acute stroke patients suffer from hyperglycemia, which is associated with a poorer prognosis and outcome [7, 9, 11]. Moreover, comorbidities themselves may exert a detrimental impact on treatment efficacy [27]. For this reason, it is important to introduce comorbidities in the experimental animal stroke model to mimic the patient’s situation and to test the efficacy of new treatments, avoiding pitfalls in translational research [27].

The exacerbated damage from hyperglycemia is typically observed in animal ischemic stroke models [10]. Our results are consistent with previous experimental data in which, animals with ischemic stroke and hyperglycemia tended to have poorer neurological recovery and increased ischemic infarct size compared to ischemic controls [10, 28–31]. Additionally, in our study, hyperglycemia enhanced diffusion coefficients. High rADC values are associated with loss of cell membrane integrity, apoptosis, shrinkage and tissue liquefaction necrosis at 30 days post-stroke [24], suggesting that higher diffusion coefficients represent poorer anatomical tissue preservation, without affecting the number of surviving neurons in the present study. Preclinical studies have demonstrated the safety and efficacy of AD-MSCs in animal models of ischemic stroke [5, 32–34]. However, whether hyperglycemia has an impact on therapeutic response has not yet been studied. In the present study, we demonstrated that the rats treated with hAD-MSCs achieved a good behavioral recovery without reducing lesion size compared with the nontreated hyperglycemic group. In line with our results, previous animal studies have reported that post-stroke cell therapy in type 2 diabetic rats promotes neurological functional outcome without affecting infarction volume [35–37]. However, in the present study, the rADC values were significantly lower in the group that received hAD-MSCs at the chronic phase. It has been described that low rADC levels are related to reduced nerve cell damage and promoting the repair of axon and myelin on the 30th day post-stroke [24]. In this sense, we have shown that hAD-MSCs administration significantly increased the number of multipolar motor neurons in the cortex, suggesting that treatment protected neuronal cells, indicating better tissue preservation post-stroke.

Ischemic injury has been related to reactive astrocytes [38, 39]. However, it has been reported that diabetic rats show inhibited astrocyte activation after ischemic stroke [39]. Conversely, in our study, GFAP levels were increased in hyperglycemic stroke rats. This controversy is probably due to differences between the diabetes model (only streptozotocin) and the stroke model used (intraluminal filament and exsanguination) compared with our study. We also detected a reduction in the glial marker GFAP after hAD-MSC administration, in accordance with previous studies [38, 40].

Inflammatory responses play an important role in post-stroke recovery. Although mild to moderate inflammation can be beneficial to brain repair, exacerbated inflammation can impede recovery and create an inhospitable environment for brain repair. In accordance with earlier studies [31, 41], we found an increase in the levels of microglia marker in hyperglycemic pMCAO rats compared with non-hyperglycemic rats. In addition, we observed that the cell treatment decreased the expression of the microglia marker.

We evaluated the expression of doublecortin and synaptophysin in the peri-infarct zone to study the role of hyperglycemia in neurogenesis and synaptogenesis. Previous studies have indicated that long-term hyperglycemia suppresses the proliferation of hippocampus neural stem/progenitor cells (NSPCs) [42–44], whereas other researchers have found an enhanced proliferation of NSPCs [45, 46]. Other authors have demonstrated that severe (20 mM) instead of mild (10 mM) hyperglycemia exacerbates ischemic injury and inhibits stroke-induced subventricular zone neurogenesis [47] and that diabetes leads to greater post-stroke spine loss which could indicate a decrease in synapses [48]. In our study, however, hyperglycemia had no effects on neurogenesis and synaptogenesis. The discrepancy among studies could be attributed to the different models used to induce hyperglycemia and stroke (transient and permanent). The efficacy of stem cell treatment on neurogenesis has been investigated by a number of studies, in which human marrow stromal cell administration increases doublecortin density post-stroke in diabetic rats [36]; however, we found that the administration of hAD-MSCs at 48 h post-stroke had no effect on the levels of doublecortin marker. Instead, our results regarding synaptophysin, a marker related to synaptic plasticity, are consistent with some articles that showed similar levels of synaptophysin between diabetic animals with and without bone marrow stromal cells [49].

Hyperglycemia and diabetes play an important role in the pathogenesis of vascular complications. We evaluated the expression of several vascular markers (CD31, collagen-IV, and α-SMA) in pMCAO model in rats. Experimental animal models in transient stroke have demonstrated increased cerebral ischemia–reperfusion-induced blood–brain barrier (BBB) disruption and neurovascular damage [11, 50–52]. Consistent with these studies, the rats with hyperglycemia showed significantly increased arterial wall thickness compared with the non-hyperglycemic group. However, our results showed that hyperglycemia did not affect the expression of CD31 or collagen-IV, perhaps because we used a permanent model instead of a transient one, contributing to exacerbated neurovascular injury as a consequence of the reperfusion. Finally, when we evaluated the effect of cell therapy, hAD-MSCs only had a significant effect on the decrease in arterial wall thickness, but it had no effect on the expression of the other markers analyzed in the study.

The precise mechanism of the therapeutic action of MSCs remains unclear. Many molecular and cellular mechanisms such as enhanced endogenous neurogenesis, trophic factor secretion, cell replacement, the formation of biobridges, and more have been suggested but not fully documented [53]. In order to elucidate these mechanisms, previous studies from our laboratory analyzed the migration and implantation of MSC when they were administered intravenously. MSC were magnetically labeled using Endorem™ (superparamagnetic iron oxide) and with a lipophilic agent using DiI and their biodistribution was analyzed in vivo by magnetic resonance [18] and after sacrifice by immunofluorescence [18, 54]. MSC were not observed in the brain, but they were found in peripheral organs (liver, lung and spleen). Even from the distance, in both studies MSC showed efficacy in promoting brain repair. These results suggest that MSC does not need to reach the brain to exert recovery after a stroke. These results agrees with other studies where estimated that only 1% of the injected cells were detected in the brain after i.v. administration of MSC [55] and that most of these cells had accumulated in internal organs such as the lungs, liver, and spleen [25, 56]. In this regards, transient early lung trapping was observed after intravenous administration of MSC radiolabeled with 370 MBq of 99mTc the HMPAO and visualized by Whole-Body Nuclear Imaging. In this study, some MSC seems to be able to migrate to the ischemic brain lesion and are eliminated by kidneys [57]. These data suggest that administered MSC have the potential to enhance brain repair mechanisms from the distance by releasing products such as trophic factors, immunomodulatory molecules, and even extracellular vesicles. Therefore, preclinical data is still needed to understand the mechanism of action of these cells to better interpret their therapeutic effects.

Regarding cryopreservation, there is an ongoing debate whether cell cryopreservation, being mandatory in a clinical scenario, compromises the cell’s therapeutic impact [58]. It seems that the cryopreservation of cells might play a role [59]. Future preclinical studies should be conducted with fresh cells to study whether their therapeutic effect could be affected by this cryopreservation.

Thus, the present data on permanent ischemia models highlights novel mechanisms how hyperglycemia leads to a worse behavioral outcome, establishing a basis for further studies investigating therapies effects comorbid conditions.

## Conclusions

In conclusion, our data suggest that rats with hyperglycemic ischemic stroke exhibit an increased lesion size and impaired brain repair processes, which likely contribute to exacerbated behavioral impairments. In addition, hAD-MSC treatment showed better anatomical tissue preservation with a decrease in the inflammatory response and in the thickness of the arterial wall, together with the improved behavioral outcome.

## Additional file

Additional file 1: **Figure S1.** Immunohistochemistry study Delimitation of the perilesional tissue with microtubule-associated protein 2 (MAP-2) and glial fibrillary acidic protein (GFAP) labeling by immunohistochemistry. (TIF 1038 kb)

## Data Availability

The original data are available from the corresponding author on request.

## Abbreviations

ADC: Apparent diffusion coefficient
AD-MSCs: Adipose tissue-derived mesenchymal stem cells
BBB: Blood–brain barrier
DAPI: 4′,6-Diamidino-2-phenylindole
DWI: Diffusion-weighted imaging
GFAP: Glial fibrillary acidic protein
H&E: Hematoxylin and eosin
hAD-MSCs: Human adipose tissue-derived mesenchymal stem cells
MRI: Magnetic resonance imaging
NSPCs: Neural stem/progenitor cells
pMCAO: Permanent middle cerebral artery occlusion
rADC: Relative apparent diffusion coefficient
SD: Standard deviation
STEPS: Stem Cell Therapies as an Emerging Paradigm in Stroke
tPA: tisular Plasminogen Activator
α-SMA: α-Smooth muscle actin
