# Supplementary figures and images for: Intravenous delivery of adipose tissue-derived mesenchymal stem cells improves brain repair in hyperglycemic stroke rats
Source: Stem Cell Res Ther. 2019 Jul 17;10:212. doi: 10.1186/s13287-019-1322-x (PMC6637493; doi:10.1186/s13287-019-1322-x)

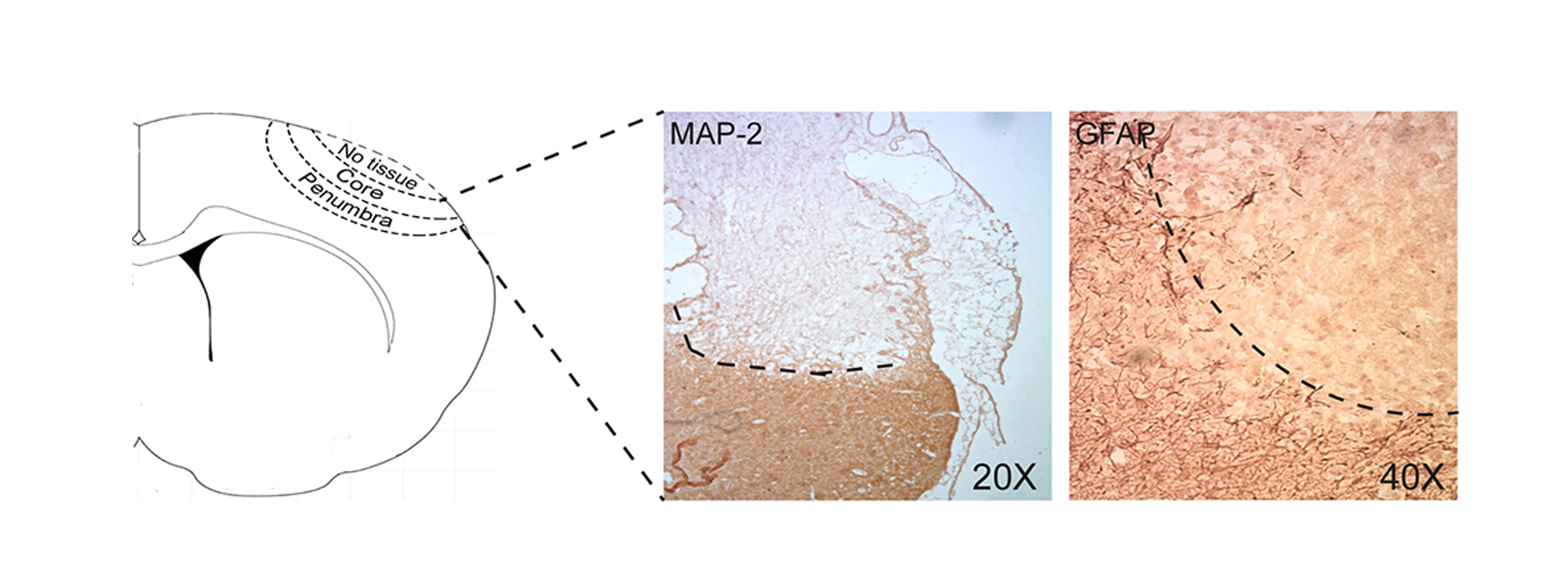

Supplement: Supplementary file 1 — Figure S1. Immunohistochemistry study Delimitation of the perilesional tissue with microtubule-associated protein 2 (MAP-2) and glial fibrillary acidic protein (GFAP) labeling by immunohistochemistry. (TIF 1038 kb) [file 13287_2019_1322_MOESM1_ESM.tif]
